# A prognostic nomogram based on LASSO Cox regression in patients with alpha-fetoprotein-negative hepatocellular carcinoma following non-surgical therapy

**DOI:** 10.1186/s12885-021-07916-3

**Published:** 2021-03-08

**Authors:** Dongdong Zhou, Xiaoli Liu, Xinhui Wang, Fengna Yan, Peng Wang, Huiwen Yan, Yuyong Jiang, Zhiyun Yang

**Affiliations:** 1 Center for Integrative Medicine, Beijing Ditan Hospital, Capital Medical University, No. 8 Jing Shun East Street, Beijing, 100015 People’s Republic of China; 2 First Clinical Medical College, Beijing University of Chinese Medicine, Chaoyang District Beijing, 100029 People’s Republic of China

**Keywords:** Alpha-fetoprotein-negative hepatocellular carcinoma, Nomogram, Prognosis, LASSO cox regression, Non-surgical therapy

## Abstract

**Background:**

Alpha-fetoprotein-negative hepatocellular carcinoma (AFP-NHCC) (< 8.78 ng/mL) have special clinicopathologic characteristics and prognosis. The aim of this study was to apply a new method to establish and validate a new model for predicting the prognosis of patients with AFP-NHCC.

**Methods:**

A total of 410 AFP-negative patients with clinical diagnosed with HCC following non-surgical therapy as a primary cohort; 148 patients with AFP-NHCC following non-surgical therapy as an independent validation cohort. In primary cohort, independent factors for overall survival (OS) by LASSO Cox regression were all contained into the nomogram1; by Forward Stepwise Cox regression were all contained into the nomogram2. Nomograms performance and discriminative power were assessed with concordance index (C-index) values, area under curve (AUC), Calibration curve and decision curve analyses (DCA). The results were validated in the validation cohort.

**Results:**

The C-index of nomogram1was 0.708 (95%CI: 0.673–0.743), which was superior to nomogram2 (0.706) and traditional modes (0.606–0.629). The AUC of nomogram1 was 0.736 (95%CI: 0.690–0.778). In the validation cohort, the nomogram1 still gave good discrimination (C-index: 0.752, 95%CI: 0.691–0.813; AUC: 0.784, 95%CI: 0.709–0.847). The calibration curve for probability of OS showed good homogeneity between prediction by nomogram1 and actual observation. DCA demonstrated that nomogram1 was clinically useful. Moreover, patients were divided into three distinct risk groups for OS by the nomogram1: low-risk group, middle-risk group and high-risk group, respectively.

**Conclusions:**

Novel nomogram based on LASSO Cox regression presents more accurate and useful prognostic prediction for patients with AFP-NHCC following non-surgical therapy. This model could help patients with AFP-NHCC following non-surgical therapy facilitate a personalized prognostic evaluation.

## Background

Hepatocellular carcinoma (HCC) is the sixth-most commonly diagnosed cancer and the fourth leading cause of tumor-related death worldwide in 2018 [1]. Accumulated evidence demonstrates that inefficient diagnosis of HCC is still a major cause of high mortality, especially in patients harboring early or small HCC [2]. Since the identification of alpha-fetoprotein (AFP) in 1970s, it has been the only serologic marker that is widely used for the HCC diagnosis [3]. For decades, HCC screening relied primarily on ultrasound imaging and AFP. Due to technical limitations, ultrasound images are often unrecognizable for HCC nodules less than 1 cm [4]. AFP is the most important and traditional serological diagnostic indicator for HCC, but about 30–40% of overall HCC patients have normal AFP levels (< 20 ng/ mL). This is referred to as AFP-negative hepatocellular carcinoma (AFP-NHCC) [5]. AFP-NHCC is an important type of liver cancer that currently causes many HCC patients to lose early diagnosis and treatment, especially in HCC with tumors less than 3 cm [6]. Although imaging technology has greatly improved the level of HCC detection, ultrasound images often fail to recognize small HCC nodules or distinguish malignant nodules from benign ones [5, 7], and the diagnosis rate for patients with AFP-NHCC is only 10.4% [8]. Studies have shown that patients with AFP-NHCC often have special clinicopathologic characteristics and prognosis, they have higher tumor differentiation, earlier TNM staging, smaller tumor size, and higher survival rates [9]. Therefore, it is essential to identify the independent prognostic risk factors of such patients, and construct prognostic prediction models.

Many staging systems have been employed to predict how HCC patients will respond over time, such as the BCLC, ALBI, Child-Pugh and TNM staging system. However, studies have shown that the traditional staging system is flawed to varying degrees. The BCLC staging system was reported to have the greatest potential in predicting patients with AFP-NHCC [10]. However, evidence has shown that the classification of the BCLC score is limited to the advanced stages of HCC [11]. Child-Pugh does not consider tumor-related factors, which are important for the prognosis of HCC patients [12], as the prognosis of HCC patients, tumor-related factors are crucial. ALBI model incorporates few factors, clinical indicators are easily available and easy to apply, but there are no tumor-related indicators to evaluate [13], as a model to evaluate the prognosis of HCC needs to be validated in a multicenter large sample. The TNM staging system is easy to use and is considered to be the best staging system for solid tumors, but its effect on the staging and prognosis of HCC is debatable because it only considers tumor characteristics but not liver function, which usually plays an important role in the prognosis of HCC patients [14]. Furthermore, these systems are not specifically designed to predict outcomes of patients with AFP-NHCC. Recently, nomograms for prediction of survival and recurrence of patients with AFP-NHCC after radical resection have been developed in two previous studies [15, 16], respectively. However, patients with AFP-NHCC with transarterial chemoembolization (TACE) and radiofrequency ablation (RFA) were not included in both studies. At the same time, there are many ways to establish independent risk factors of prognostic prediction model.

LASSO Cox regression is a method for variable selection and shrinkage in Cox proportional hazards model, proposed by Tibshirani et al. in 1997 [17]. LASSO Cox regression analysis constructs a penalty function to obtain a more refined model. And a number of studies have shown that it plays an important role in cancer research: Li et al. applied LASSO Cox regression to the establishment of a prognostic model for lung adenocarcinoma [18]; Xiong et al. establishment of an outcome model for bladder cancer [19]; Jiang et al. establishment of a prognostic model gastric cancer [20]; Wu et al. establishment of a prognostic model pancreatic cancer [21]. Meanwhile, Liu et al. applied LASSO Cox regression to the establishment of a prognostic model for hepatocellular carcinoma [22, 23]. However, the above studies all used LASSO Cox screening genes, and there were few reports on the screening of clinical indicators.

Therefore, the aim of our study was applying a new method to establish and validate a new model that combines clinical pathological factors, biochemical indicators, for predicting the prognosis of patients with AFP-NHCC. In addition, a comparison between the constructed nomograms and traditional staging systems was conducted to determine whether the nomograms provided more accurate prediction in prognosis.

## Methods

### Patient selection

We performed a retrospective cohort study on 558 AFP-negative (< 8.78 ng/mL) HCC patients following non-surgical therapy using data from the Beijing Ditan Hospital between January 2008 and December 2016. The HCC diagnosis data included biopsy, radiology. First, we selected patients based on hepatic angiography, pathology in combination with ultrasonography, computed tomography (CT), and magnetic resonance imaging (MRI). Next, we only included patients with complete clinical data. Our exclusion criteria included: (1) other viral infections such as human immunodeficiency virus (HIV); (2) metastatic liver cancer; (3) pregnant women; (4) incomplete data; (5) following surgical therapy; and (6) patients with liver transplantation and survival time < 15 days. The primary cohort of our study included 410 clinically diagnosed patients with AFP-NHCC, retrospectively studied via an information system between January 2008 and December 2014. The patients were followed for 5 years (death follow-up stopped) and first hospitalization records were kept. One hundred forty eight patients with AFP-NHCC between January 2015 and December 2016 as an independent validation cohort. The patients were followed for 3 years (death follow-up stopped) and first hospitalization records were kept. It is recommended that all HCC patients undergo regular follow-up visits according to clinical guidelines after completion of hospital admission, usually every 3 months for the first 2 years and once a year for the next 3 to 5 years. Patients who did not come to our hospital on time for review were given treatment information and living conditions by telephone follow-up (telephone follow-up by our clinicians), the last follow-up occurred in December 2019. The outcome of our study was overall survival (OS), defined as the time from the diagnosis of HCC to the last follow-up or death. The study was approved by the ethics committee of Beijing Ditan Hospital, Capital Medical University and was conducted in accordance with the standards of the Declaration of Helsinki.

### Laboratory measurements

Patients are routinely examined at the first visit. Data provided include: sex, age, tumor multiplicity, tumor size, ascites, cirrhosis, etiology, portal vein tumor thrombus (PVTT), neutrophil to lymphocyte ratio (NLR), hemoglobin (HGB), platelet (PLT), creatinine (CR), alanine aminotransferase (ALT), aspartate aminotransferase (AST), total bilirubin (TBIL), albumin (ALB), gamma-glutamyl transpeptidase (γ-GGT), prothrombin time activity (PTA), lactate dehydrogenase (LDH), carbohydrate antigen 199 (CA199), C reactive protein (CRP), carcino-embryonic antigen (CEA), AFP. When laboratory values at the time of HCC diagnosis were higher than clinical normal, it was classified as elevated. The tumor was staged using the BCLC staging system and liver function was scored using Child-Pugh.

### Statistical analysis

We convert continuous variables into classification variables, which makes the model more objective and simpler. The cut-off value of the classification variable was the normal value of clinical laboratory examination. The cut-off value of numeric values as follows: HGB was 120 g/L, PLT was 100*10^9/L, CR was 111μmoI/L, ALT was 50 U/L, AST was 40 U/L, TBIL was 18.8 μmol/L, ALB was 40 g/L, LDH was 250 U/L, GGT was 60 U/L, PTA was 70%, CEA was 5 ng/ml, CA199 was 37u/ml, CRP was 5 mg/L. At the same time, it is consistent with the cut-off value of previous literatures published by our team [24]. The cut-off value of NLR was 5 determined according to relevant literature [25, 26]. In this study, we used the primary cohort to plot nomogram1 based on LASSO Cox regression, and used the primary cohort to plot nomogram2 based on Forward Stepwise Cox regression. Based on the established nomogram1 and nomogram2, the C-index and calibration curves were derived based on Cox regression analysis, NRI and IDI scores were performed, and decision curves were plotted. Nomogram1 was found to be superior to nomogram2 in the primary and validation cohorts. The total score for each patient was calculated based on nomogram 1, and three groups of patients with high, medium, and low prognostic risk (based on the total score) were divided according to interquartile values. The Kaplan-Meier curve was applied in MedCalc software, and the risk group was compared using the three-point factor, log-rank test, and the two-tailed *P* value < 0.05 was statistically significant.

Statistical analysis was done using SPSS 24.0 (IBM, Chicago, IL, USA), R software (version 3.6.3; http://www.Rproject.org) and MedCalc19.2.0. Categorical variables were classified based on clinical findings. SPSS 24.0 was used to perform the Forward Stepwise Cox regression. R version 3.6.3 for “foreign” package, “survival” package, and “rms” package, were used to plot nomograms and calibration plots, and calculate C-index; “glmnet” package was used to perform the LASSO Cox regression; “nricens” package for NRI calculation; “stdca” package for decision curve; “time ROC” package for time-dependent ROC curve. MedCalc19.2.0 for low-risk group, middle-risk group and high-risk group Kaplan-Meier curve plotting.

## Results

### Basic characteristics

In the primary cohort, 410 patients with AFP-NHCC met the inclusion and exclusion criteria and were included in this study. A total of 148 patients with AFP-NHCC were included in the validation cohort. The clinical characteristics of patients in the two independent cohorts are shown in Table 1. Most of the study population was male (79.8% vs 83.8%; *p* = 0.286), and the patients were older than 50 years (77.8% vs 83.1%; *p* = 0.173) in the primary and validation cohort. In addition, (78.5% vs 81.1%; *p* = 0.876) of the patients presented positive HBsAg. Most patients remained at BCLC stage 0-B (78.5% vs 80.4%; *p* = 0.632) and Child-Pugh A-B (87.1% vs 92.6%; *p* = 0.168), and (24.1% vs 18.2%; *p* = 0.212) had a tumor size ≥5 cm (Table 1). In the primary cohort, 224 patients died from cancer within 5 years.

**Table 1:** Basal clinicopathologic characteristics in training and validation cohort

	Training set	Validation set	
Characters	*n* = 410(%)	*n* = 148(%)	*P*
Sex			
Male	327(79.8)	124 (83.8)	0.286
Female	83 (20.2)	24 (16.2)	
Age			
<50	91 (22.2)	25 (16.9)	0.173
≥ 50	319 (77.8)	123 (83.1)	
Tumor size(cm)			
<3	230 (56.1)	84 (56.8)	0.212
3–5	81 (19.8)	37 (25.0)	
≥ 5	99 (24.1)	27 (18.2)	
Tumor multiplicity			
Single	266 (64.9)	77 (52.0)	0.006
Multiple	144 (35.1)	71 (48.0)	
Cirrhosis			
Yes	377 (92.0)	135 (91.2)	0.780
No	33 (8.0)	13 (8.8)	
PVTT			
Yes	145 (35.4)	40 (27.0)	0.065
N0	265 (64.6)	108 (73.0)	
Ascites			
Yes	162 (39.5)	53 (35.8)	0.428
No	248 (60.5)	95 (64.2)	
HBsAg			
Positive	330 (78.5)	120 (81.1)	0.876
Negative	80 (21.5)	28 (18.9)	
NLR			
<5	344 (83.9)	132 (89.2)	0.119
≥ 5	66 (16.1)	16 (10.8)	
HGB(g/L)			
<120	159 (38.8)	58 (39.2)	0.930
≥ 120	251 (61.2)	90 (60.8)	
PLT(*10^9/L)			
<100	219 (53.4)	80 (54.1)	0.894
≥ 100	191 (46.6)	68 (45.9)	
CR (μmoI/L)			
<111	391 (95.4)	141 (95.3)	0.962
≥ 111	19 (4.6)	7 (4.7)	
ALT(U/L)			
<50	347 (84.6)	130 (87.8)	0.343
≥ 50	63 (15.4)	18 (12.2)	
AST(U/L)			
<40	268 (65.4)	113 (76.4)	0.014
≥ 40	142 (34.6)	35 (23.6)	
TBIL (μmol/L)			
<18.8	218 (53.2)	87 (58.8)	0.240
≥ 18.8	192 (46.8)	61 (41.2)	
ALB(g/L)			
< 40	275 (67.1)	103 (69.6)	0.574
≥ 40	135 (32.9)	45 (30.4)	
LDH(U/L)			
<250	365 (89.0)	131 (88.5)	0.865
≥ 250	45 (11.0)	17 (11.5)	
GGT (U/L)			
<60	273 (66.1)	99 (66.9)	0.861
≥ 60	139 (33.9)	49 (33.1)	
PTA (%)			
< 70	142 (34.6)	42 (28.4)	0.165
≥ 70	268 (65.4)	106 (71.6)	
CEA (ng/ml)			
< 5	345 (84.1)	113 (76.4)	0.034
≥ 5	65 (15.9)	35 (23.6)	
CA199(u/ml)			
< 37	364 (89.3)	133 (90.5)	0.664
≥ 37	44 (11.7)	14 (9.5)	
CRP (mg/L)			
< 5	266 (64.9)	99 (71.2)	0.180
≥ 5	144 (35.1)	40 (28.8)	
Child-Pugh			
A	230 (56.1)	92 (62.2)	0.168
B	127 (31.0)	45 (30.4)	
C	53 (12.9)	11 (7.4)	
BCLC stage			
0-B	322 (78.5)	119 (80.4)	0.632
C-D	88 (21.5)	29 (19.6)	
ALBI grade			
I	149 (36.3)	76 (51.4)	0.004
II	207 (50.5)	59 (39.9)	
III	56 (13.7)	13 (8.8)	
TNM			
I	159 (38.8)	53 (35.8)	0.005
II	66 (16.1)	42 (28.4)	
III	131 (32.0)	43 (29.1)	
IV	54 (13.2)	10 (6.8)	

### Biomarker selection

**Fig. 1:**
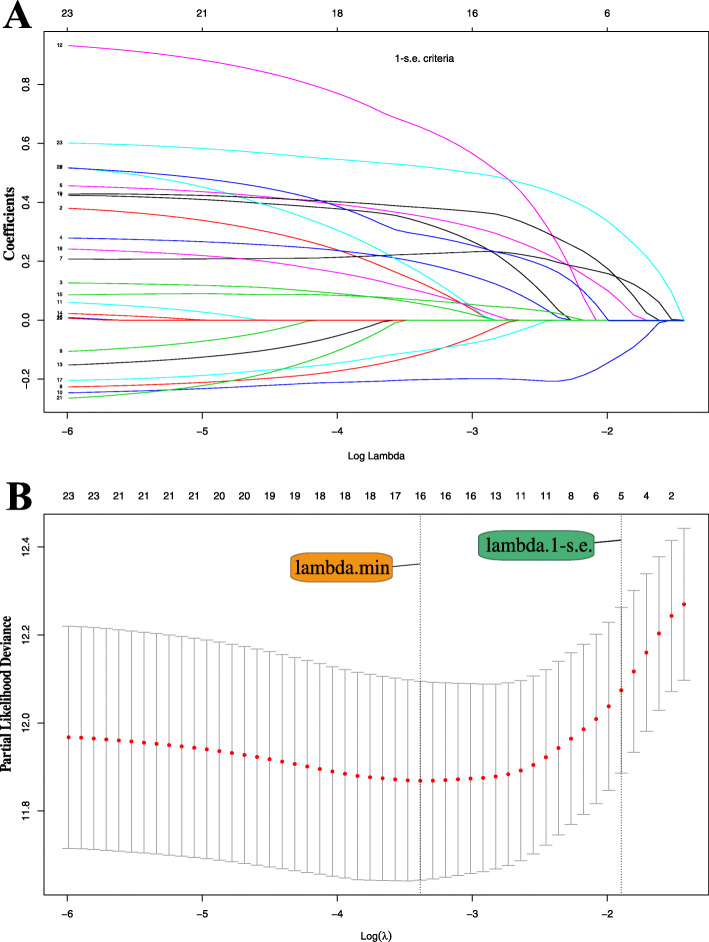
**a** LASSO coefficient profiles of the 23 risk factors. **b** Five risk factors selected using LASSO Cox regression analysis. The two dotted vertical lines were drawn at the optimal scores by minimum criteria and 1-s.e. criteria (At minimum criteria including Sex, Age, Tumor size, Tumor number, Cirrhosis, PVTT, Ascites, HBV, HGB, CR, AST, ALB, LDH, γ-GGT, CA199 and CRP; At 1-s.e. criteria including PVTT, Ascites, HGB, γ-GGT and CRP)

All available clinical indicators, including clinicopathological features and biomarkers (Table 1), were subjected to LASSO Cox regression, with a significant correlation between sex, age, tumor size, tumor number, cirrhosis, PVTT, ascites, HBV, HGB, CR, AST, ALB, LDH, γ-GGT, CA199, CRP and OS at minimum values (Fig. 1a). Further disciplinary regression was performed to take 1-s.e. criteria PVTT, ascites, HGB, γ-GGT, CRP as independent risk factors for prognosis in patients with AFP-NHCC (Fig. 1b). Inclusion of all clinical indicators in Cox univariate analysis, there was a significant correlation with OS in sex, tumor size, tumor multiplicity, cirrhosis, PVTT, ascites, HBsAg, NLR, HGB, PLT, CR, AST, TBIL, ALB, LDH, γ-GGT, PTA, CA199, and CRP. Cox multivariate analysis was then performed to identify the factors that were distinguished in the Cox univariate analysis. The results showed that sex, PVTT, CR, γ-GGT, and CRP were independent risk factors for prognosis in patients with AFP-NHCC (Table 2).

**Table 2:** Univariate and multivariate cox hazards analysis of the training cohort

	Univariate analysis		Multivariate analysis	
Characteristic	HR (95% CI)	P	HR (95% CI)	p
Sex				
Male/Female	1.577 (1.163–2.138)	0.003	1.655 (1.191–2.300)	0.003
Tumor size(cm)				
<3/3–5/≥5	1.274 (1.093–1.484)	0.002		
Tumor multiplicity				
Single/Multiple	1.514 (1.160–1.976)	0.002		
Cirrhosis				
Yes/N0	2.265 (1.201–4.271)	0.012		
PVTT				
Yes/N0	2.052 (1.497–2.811)	<0.001	1.547 (1.162–2.059)	0.003
Ascites				
Yes/N0	2.314 (1.779–3.010)	<0.001	1.304 (0.918–1.852)	0.138
HBsAg				
Positive/Negative	0.622 (0.459–0.844)	0.002		
NLR				
<5, ≥5	1.723 (1.246–2.384)	0.001		
HGB(g/L)				
<120, ≥120	0.447 (0.343–0.581)	<0.001	0.785 (0.568–1.084)	0.142
PLT(*10^9/L)				
<100,≥100	0.690 (0.503–0.948)	0.022		
CR (μmoI/L)				
<111, ≥111	3.486 (2.116–5.745)	<0.001	2.200 (1.254–3.858)	0.006
AST(U/L)				
<40, ≥40	1.908 (1.463–2.489)	<0.001		
TBIL (μmol/L)				
<18.8, ≥18.8	1.602 (1.232–2.084)	<0.001		
ALB(g/L)				
< 40, ≥40	0.468 (0.342–0.639)	<0.001		
LDH(U/L)				
<250, ≥250	1.993 (1.389–2.861)	<0.001		
GGT (U/L)				
<60, ≥60	2.147 (1.647–2.799)	<0.001	1.495 (1.087–2.056)	0.013
PTA(%)				
< 70, ≥70	0.574 (0.440–0.748)	<0.001		
CA199(u/ml)				
<37, ≥37	2.479 (1.738–3.534)	<0.001		
CRP (mg/L)				
<5, ≥5	2.545 (1.955–3.313)	<0.001	1.823 (1.341–2.478)	<0.001

### Development the prediction model

**Fig. 2:**
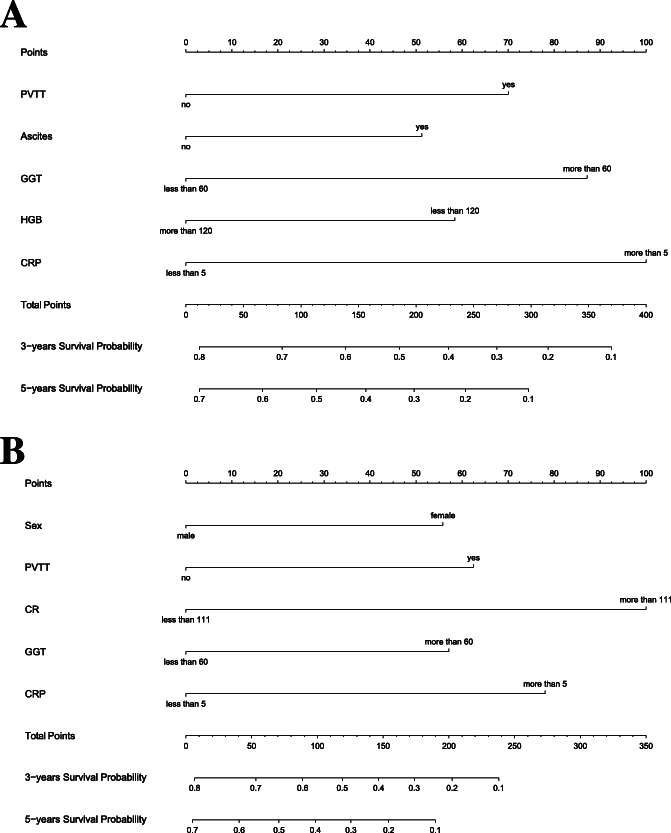
**a** Nomogram1 including PVTT, Ascites, γ-GGT, HGB and CRP, for three- and five-years overall survival (OS) in patients with AFP-negative HCC. **b** Nomogram2 including Sex, PVTT, CR, γ-GGT and CRP, for three- and five-years overall survival (OS) in patients with AFP-negative HCC. The nomogram1 and nomogram2 are valued to obtain the probability of three- and five-years survival by adding up the points identified on the points scale for each variable

Nomogram1 and nomogram2 were constructed to predict 3 and 5-year OS based on prognostic factors determined by both instruments (Fig. 2). Nomogram1 and nomogram2 used consistency index (C-index), AUC and time-dependent ROC curves in the primary cohort, respectively, and the calibration curves were plotted. The C-index of nomogram1 in the primary cohort was 0.708 (95% CI: 0.673–0.743); the AUC (ROC curve) was 0.736 (95%CI: 0.690–0.778), with sensitivity (62.05%), specificity (76.34%), PPV (76.0%) and NPV (62.6%). The C-index of nomogram2 was 0.706 (95%CI: 0.673–0.739); the AUC was 0.714 (95%CI: 0.667–0.757), with sensitivity (74.55%), specificity (61.83%), PPV (70.2%) and NPV (66.9%). Continuity cut point 0.05NRI (− 0.037), subtype cut point 0.5NRI (− 0.037), IDI: 0.006 (*p* = 0.29). We get similar results in the validation cohort. All suggest that nomogram1 is better than nomogram2.

### Performance of the nomogram

**Fig. 3:**
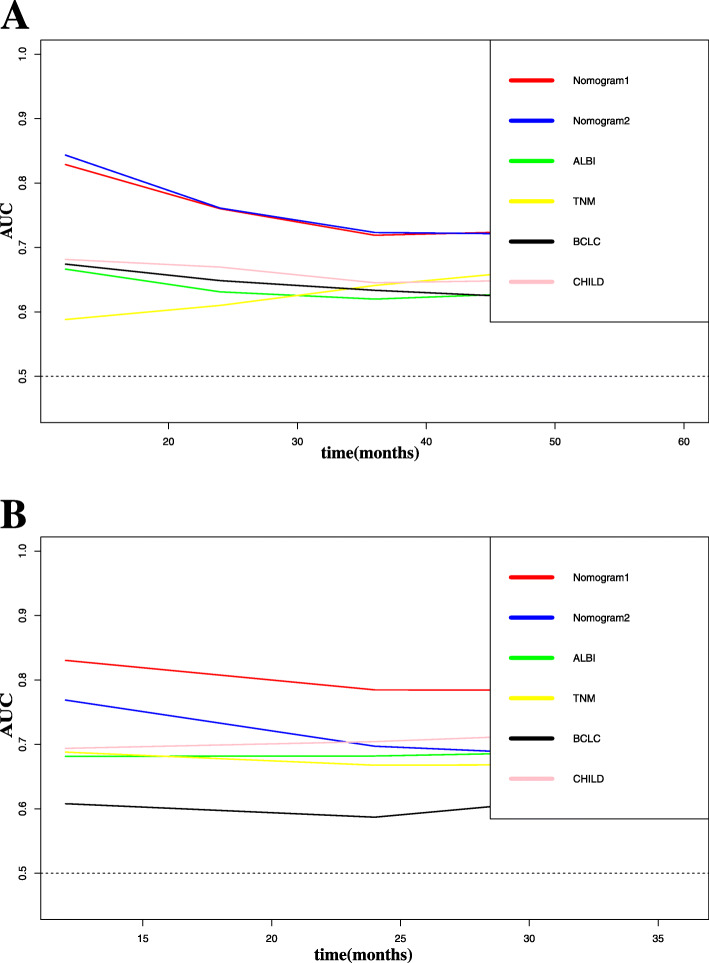
Time-ROC curve of the six models in the primary and validation cohort. Red line: Nomogram1.Blue line: Nomogram1.Green line: ALBI. Yellow line: TNM. Black line: BCLC. Pink line: CHILD. **a** Time-ROC curve of the six models in the primary. **b** Time-ROC curve of the six models in the validation cohort

**Fig. 4:**
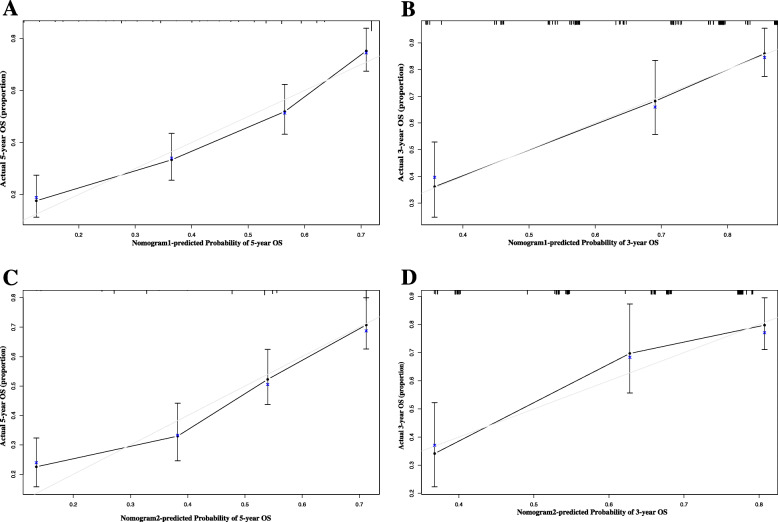
Calibration curve of the nomogram1 and nomogram2 in the primary and validation cohort, with the x-axes are actual survival estimated by the nomogram, the y-axes are observed survival calculated by the Kaplan-Meier method. **a** Five-year survival OS in the primary cohort- nomogram1. **b** Three-year OS in the validation cohort- nomogram1. **c** Five-year survival OS in the primary cohort- nomogram2. **d** Three-year OS in the validation cohort- nomogram2

In the primary cohort, the C-index (0.708) and AUC (0.736) of nomogram1 outperformed the nomogram2 and the other models (Table 3). Time-dependent ROC curves suggest that nomogram 1 and nomogram 2 are similar, but significantly better than traditional modes such as Child-Pugh, BCLC, ALBI, TNM (Fig. 3a). Calibration plots for 3 and 5-year OS probabilities show the best agreement between the nomogram1 predictions and actual observations (Fig. 4a, c). In the validation cohort, the C-index (0.752) and AUC (0.784) of nomogram1 outperformed the nomogram2 and the other models (Table 3). The time-dependent ROC curve suggests nomogram1 is significantly superior to nomogram2, Child-Pugh, BCLC, ALBI and TNM staging systems (Fig. 3b). Calibration plots for 3-year OS probabilities show the best agreement between nomogram1 predictions and actual observations (Fig. 4b, d).

**Table 3:** C-index and AUC of prognostic staging systems for Training and Validation cohort

	Training cohort				Validation cohort			
Models	C-index	95% CI	AUC	95% CI	C-index	95% CI	AUC	95% CI
Nomogram1	0.708	0.673–0.743	0.736	0.690–0.778	0.752	0.691–0.813	0.784	0.709–0.847
Nomogram2	0.706	0.673–0.739	0.714	0.667–0.757	0.714	0.647–0.781	0.677	0.595–0.751
ALBI	0.616	0.583–0.649	0.659	0.611–0.705	0.664	0.593–0.735	0.691	0.610–0.764
TNM	0.606	0.573–0.639	0.656	0.607–0.702	0.649	0.578–0.720	0.669	0.587–0.744
BCLC	0.609	0.582–0.636	0.628	0.579–0.674	0.608	0.547–0.669	0.631	0.547–0.708
CHILD	0.629	0.598–0.660	0.672	0.625–0.718	0.677	0.612–0.742	0.722	0.643–0.793

**Fig. 5:**
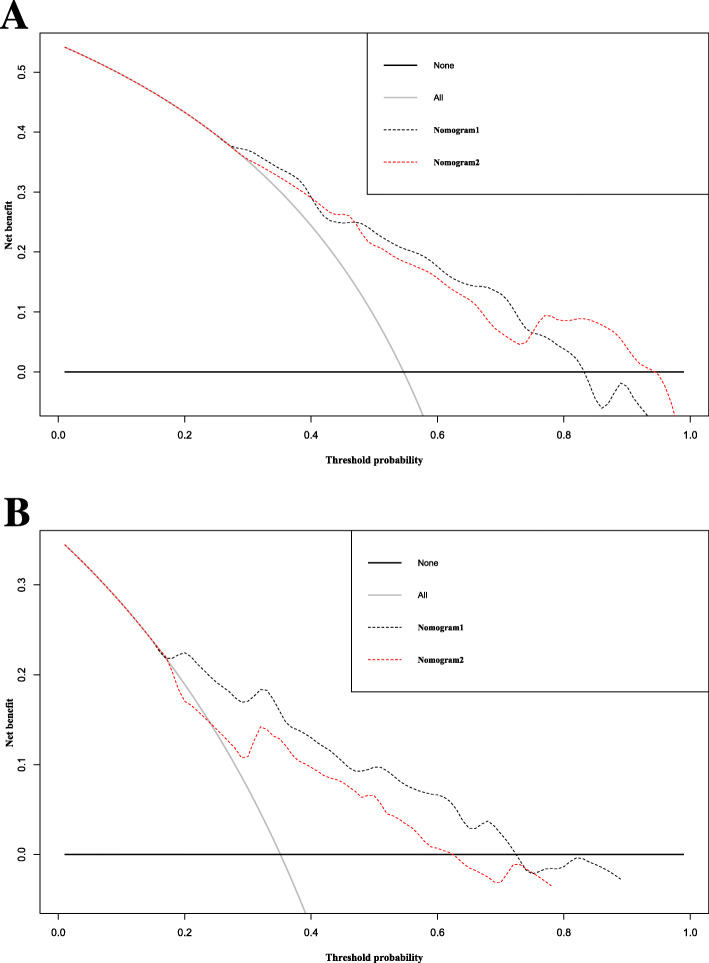
Decision curve analysis for overall survival in the primary and validation cohort. Black line: All patients dead. Gray line: None patients dead. Black dashed line: Model of nomogram1. Red dashed line: Model of nomogram2. **a** Decision curve analysis for overall survival in the primary. **b** Decision curve analysis for overall survival in the validation cohort

The decision curve analysis of nomogram 1 and nomogram 2 is also meaningful (Fig. 5). In the primary cohort the cue differences were small, but in the validation cohort nomogram1 and nomogram2 were better at predicting OS than either the all-patient death scenario or the no-patient death scenario if the patient threshold probability was > 20%. Moreover, the net benefit is comparable; in this range, the predicted OS of nomogram 1 is more advantageous than nomogram 2.

### Application of the Nomogram model for risk stratification

**Fig. 6:**
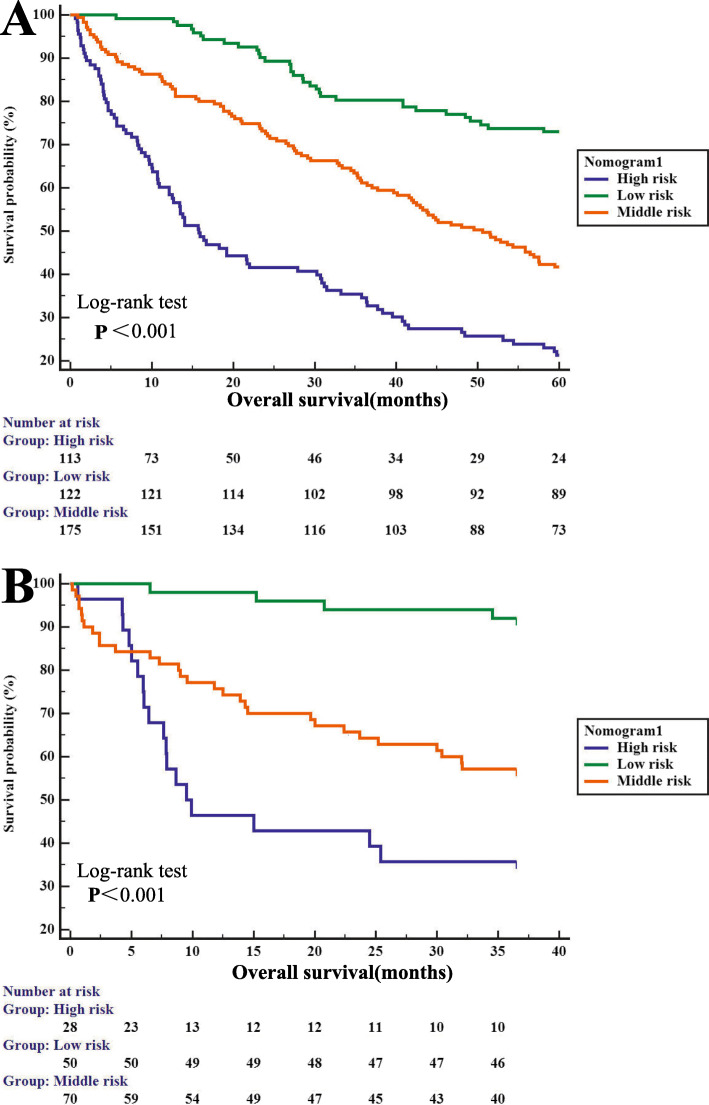
Kaplan-Meier survival curves of nomogram1. **a** In the primary cohort. **b** In the validation cohort

Based on the nomogram1 we developed in this study, we subdivided the patients into low-risk, middle-risk, and high-risk groups, and the patients with AFP-NHCC showed good prognostic classification in both the primary cohort and the validation cohort. In the primary cohort, there were 122 cases in the low-risk group, 175 cases in the middle-risk group, and 113 cases in the high-risk group. Intergroup OS was (52.111 ± 1.386) months, (40.960 ± 1.622) months, and (26.219 ± 2.138) months (*p* < 0.001) (Fig. 6a). In the validation cohort, there were 50 cases in the low-risk group, 70 cases in the middle-risk group, and 28 cases in the high-risk group. Intergroup OS was (35.121 ± 0.777) months, (25.983 ± 1.670) months, and (18.721 ± 2.685) months (*p* < 0.001) (Fig. 6b).

## Discussion

It is important to have an accurate prognosis of the tumor after relevant treatment. In this study, we identified 3 years and 5 years of independent risk factors for OS in patients with AFP-NHCC in the primary cohort, and established novel, effective, and verified nomograms1 to predict individuals at 3 years and 5 years OS. PVTT, ascites, HGB, γ-GGT and CRP have been included in the nomogram1. In addition, nomogram1 shows a higher discriminatory power, and the C-index of the two independent cohorts are 0.708 and 0.752, respectively. In the subsequent study, the calibration curve and decision curve analysis were used to assess the predicted precision and clinical utility of the nomogram1. Our model showed a better net benefit and higher consistency between the nomogram prediction and the actual observation.

Previous studies have demonstrated the prominent role of tumor burden and grade, liver function, degree of hepatic dysfunction, and performance status in the prognosis of HCC. For many years, the traditional Child-Pugh rating system is the most widely used method for assessing liver function and predicting therapeutic efficacy [27]. Our study also found that the C-index (0.629) and AUC (0.672) of Child-Pugh in the primary cohort were significantly higher than ALBI, TNM, and BCLC. We also obtained the same results in the validation cohort. However, patients with AFP-NHCC have special clinicopathologic characteristics and prognosis, they have higher tumor differentiation, earlier TNM staging, smaller tumor size, and higher survival rates [9]. Most models are built without taking into account the specificity of liver biology function in patients with AFP-NHCC and do not fully reflect an accurate prognosis. The above studies indicate that our model is more accurate for the prognosis of patients with AFP-NHCC. We found C-index (0.708), AUC (0.736) and time-dependent ROC curves of our model was better than the other models, such as Child-Pugh, BCLC, ALBI, TNM. The calibration curve for probability of OS showed good homogeneity between prediction by our model and actual observation. DCA demonstrated that our model was clinically useful. We get similar results in the validation cohort.

A variety of nomograms have been developed to predict the prognosis of certain cancers and have been shown to be more accurate than traditional staging systems. However, most of them are limited to Forward Stepwise Cox regression risk factor screening, which is not conducive to small sample size, multi-indicator model screening [28]. LASSO Cox regression analysis constructs a penalty function to obtain a more refined model. It compresses the regression coefficients (the sum of the absolute values of the mandatory coefficients is less than a fixed value) and sets some regression coefficients to 0. LASSO Cox regression analysis can not only solve the problem of over-fitting, but also extract useful features effectively [17]. Meanwhile, LASSO Cox regression is more applicable in decisions with more clinical indicators. In this study, we find that the model variables were screened by LASSO Cox regression has better accuracy and resolution than the model variables were screened by Forward Stepwise Cox regression. Number of studies have shown that it plays an important role in cancer research: especially in lung adenocarcinoma [18]; bladder cancer [19]; gastric cancer [20]; and pancreatic cancer [21]. Meanwhile, Liu et al. applied LASSO Cox regression to the establishment of a prognostic model for hepatocellular carcinoma [22, 23]. Although, the above studies all used LASSO Cox screening genes, and there were few reports on the screening of clinical indicators. In the primary cohort, we find the C-index (0.708) and AUC (0.736) of nomogram1 is superior to the C-index (0.706) and AUC (0.714) of nomogram2. We got better results in the validation cohort.

**Fig. 7:**
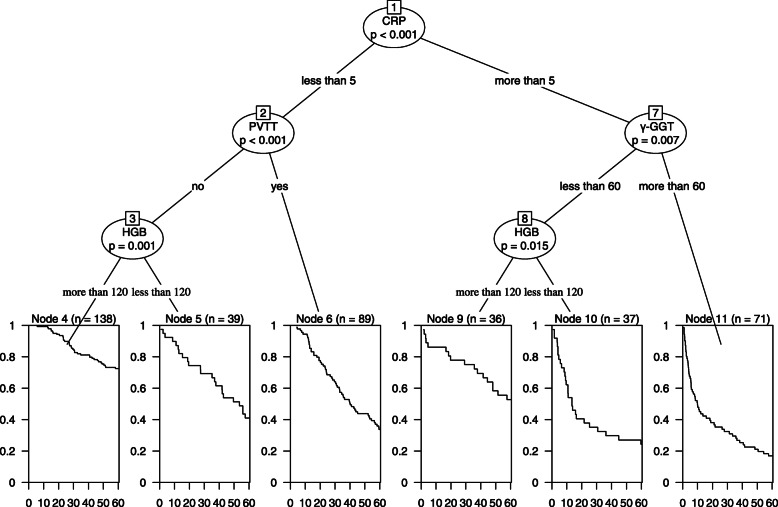
Decision Tree of nomogram1 5 years OS in the primary cohort including CRP, PVTT, γ-GGT and HGB

Our final nomogram included five independent risk factors, liver function parameters: ascites (C-index: 0.606) and γ-GGT (C-index: 0.596); inflammatory indicator: CRP (C-index: 0.596); and a tumor-related index: HGB (C-index: 0.604) and PVTT (C- index: 0.582). The importance of various risk factors can be shown by the Decision Tree (Fig. 7). CRP is an exquisitely sensitive marker of inflammation and tissue damage [29]. CRP is synthesized in the liver and is secreted into the plasma as a pentamer, belonging to the family of pentraxins, together with serum amyloid protein [30]. Based on recent studies, the serum CRP levels are correlated with the poor prognosis in HCC: Chun et al. found that CRP can predict overall survival and recurrence rates after hepatectomy in patients with HCC patients [31]; Na et al. found that CRP was independently associated with OS in non-surgical HCC patients [32]; and She et al. found that CRP is a biomarker of AFP-negative HBV-related hepatocellular carcinoma [5]. While the molecular mechanism underlying tumor-related CRP elevation in HCC or other cancers remains unknown, several possible mechanisms have been proposed. For instance, cancer growth and tumor-host cell interaction could increase CRP levels [33]. Additionally, CRP levels might reflect an inflammatory response activated as a secondary process in reaction to tumor necrosis or other local tissue damage. Moreover, cancer cells produce cytokines via autocrine pathways, such as IL-6 and IL-8, which in turn induce CRP production [34]. The significance of inflammatory signaling through the STAT3 pathway has been emphasized by numerous studies of HCC and other malignancies. PVTT is the most common form of macrovascular invasion of HCC. Multiple case series have suggested that the PVTT is a common phenomenon with a prevalence rate ranging from 10% to over 60% [35–38]. The ascites, jaundice, hepatic encephalopathy, and liver failure may induce by the formation of PVTT [39]. The presence of PVTT in patients with HCC has been consistently demonstrated by different series to be associated with poor prognoses, with a hazard ratio of death close to 2 [38, 40]. Clinically, PVTT is associated with large tumor size, increased tumor number, higher tumor grade, worse Child-Pugh class and higher serum alpha-fetoprotein (AFP). In this study, PVTT was found to be a strong prognostic indicator in patients with AFP-NHCC. It is to be verified with a large multi-center sample. As a crucial enzyme in glutathione metabolism, γ-GGT was continually elevated in metabolic-induced hepatic injury [41]. Salvatore et al. demonstrated that the level of serum γ-GGT elevated with the process of liver carcinogenesis and promoted tumor progression in an HCC animal model of male Wistar rats [42]. Serum levels of γ-GGT could also help with the selection of further treatment and clinical outcomes for patients with HCC [43]. Carr et al. found that patients with significantly high GGT values were prone to poor overall survival in cases of low AFP HCC [44]. The results of this study and previous studies indicate that the increase of γ-GGT is closely related to the prognosis of patients with AFP-NHCC [15, 45]. Anemia is common in cancer patients. HGB levels have been shown to have an impact on survival both before and during anti-cancer therapy [46]. The pre-treatment anemia in HCC patients was found to be 7.0%, which is less than the 12.8% of cancer-related anemia [47]. The causes of anemia in HCC patients include nutritional deficiencies, hemolysis, blood loss, and tumor cell infiltration of the bone marrow [48]. In addition, chronic liver injury can also lead to anemia in HCC patients [49]. It has also been shown that downregulation of iron-regulated genes, including heparin, ceruloplasmin, transferrin, and transferrin receptors, disrupts the systemic iron homeostasis, leading to anemia in patients with HCC [50]. It has been demonstrated that HGB is an independent prognostic factor in patients with HCC, which is similar to the results we obtained [47]. Ascites is the hallmark of portal hypertension [51]. Besides, ascites may also be associated with large tumor burden and vascular invasion of HCC, suggesting that the cause of ascites could be attributed to both tumoral and cirrhotic factors [52, 53]. Hence, ascites is not only an indicator of deterioration of liver functional reserve but could also be a signal of tumor progression. Studies have shown that ascites reduces long-term survival in patients with liver cancer [54]. It is similar to the results of our current study. Tumor size is one of the most important parameters of tumor burden. However, evaluation of tumor number failed to predict the prognosis of HCC patients in the present study, although other studies have demonstrated its prominent role in prognostic prediction [55]. Meanwhile, tumor markers have not been included in this study. After research and repeated comparison, our nomogram exhibited superior discrimination ability for the prediction of prognostic in patients with AFP-NHCC. Hence, our novel nomogram could be used to guide routine follow-up for patients. PVTT, ascites, HGB, γ-GGT and CRP should be given great importance in patients with AFP-NHCC. In addition, patients given a high score by the nomogram should undergo more high-end imaging examinations, such as MRI or CT exams, and the interval time of follow up should be reduced, even if the last test results have no causes for concern.

Although our nomogram performed well, several limitations need to be addressed. Firstly, patients with AFP-NHCC were limited and all came from the same hospital, and not validated internally, which may create bias in evaluating the predictive value of these markers; in the validation cohort the follow-up time was shorter, and close monitoring and five-year follow-up data are still required for patients in the validation cohort. Secondly, because the present study was a retrospective study for predicting anticipated future performance, our results need to be confirmed by prospective cohort studies. Thirdly, all patients we included are following non-surgical therapy, whether our nomogram can applicable the patients who following radical resection remains uncertain. Hence, future prospective studies require multiple centers, a larger scale, and more detailed information to validate these results.

## Conclusions

We applied new methods to develop and validate nomogram to predict the OS in patients with AFP-NHCC following non-surgical therapy. Nomogram1 presented in this study is statistically easier than previous model screening methods and more accurate than ALBI, TNM, Child-Pugh, BCLC, and provides a useful tool for prognosis. The combination of ascites, γ-GGT, CRP, HGB, and PVTT as economic, simple, effective, and promising biomarkers, possessed a high diagnostic efficiency in the progression of patients with AFP-NHCC, especially in patients following non-surgical therapy.

## Data Availability

The datasets used or analyzed during the current study are available from the corresponding author on reasonable request. In order to protect study participant privacy, our data cannot be shared openly.

## Abbreviations

HCC: Hepatocellular carcinoma
AFP-NHCC: Alpha-fetoprotein-negative hepatocellular carcinoma
OS: Overall survival
HR: Hazard ratio
95% CI: 95% confidence interval
AFP: Alpha fetoprotein
PVTT: Portal vein tumor thrombus
ALB: Albumin
ALP: Alkaline phosphatase
ALT: Alanine aminotransferase
AST: Aspartate aminotransferase
TBIL: Total bilirubin
LDH: Lactate dehydrogenase
ɣ-GGT: ɣ-Glutamyl transpeptidase
NLR: Neutrophil to lymphocyte ratio
HGB: Hemoglobin
PLT: Absolute platelet count
PTA: Prothrombin activity
RFA: Radiofrequency ablation
TACE: Transcatheter arterial chemoembolization
TNM: The Tumor, node, metastasis
CRP: C reactive protein
CEA: Carcino-embryonic antigen
CA199: Carbohydrate antigen 199
BCLC: Barcelona Clinic Liver Cancer
